# Exploring Potential Biomarkers Underlying Pathogenesis of Alzheimer’s Disease by Differential Co-expression Analysis

**Published:** 2018

**Authors:** Fereshteh Izadi, Mohammad Hasan Soheilifar

**Affiliations:** 1. Department of Genetics, Evolution and Environment, Darwin Building, University College London (UCL), London, UK; 2. Research Center for Molecular Medicine, Hamedan University of Medical Sciences, Hamedan, Iran

**Keywords:** Alzheimer’s disease, Computational biology, Dementia

## Abstract

**Background:**

Alzheimer’s Disease (AD) is the most common form of dementia in the elderly. Due to the facts that biological causes of AD are complex in addition to increasing rates of AD worldwide, a deeper understanding of AD etiology is required for AD treatment and diagnosis.

**Methods:**

To identify molecular pathological alterations in AD brains, GSE36980 series containing microarray data samples from temporal cortex, frontal cortex and hippocampus were downloaded from Gene Expression Omnibus (GEO) database and valid gene symbols were subjected to building a gene co-expression network by a bioinformatics tool known as differential regulation from differential co-expression (DCGL) software package. Then, a network-driven integrative analysis was performed to find significant genes and underlying biological terms.

**Results:**

A total of 17088 unique genes were parsed into three independent differential co-expression networks. As a result, a small number of differentially co-regulated genes mostly in frontal and hippocampus lobs were detected as potential biomarkers related to AD brains. Ultimately differentially co-regulated genes were enriched in biological terms including response to lipid and fatty acid and pathways mainly signaling pathway such as G-protein signaling pathway and glutamate receptor groups II and III. By conducting co-expression analysis, our study identified multiple genes that may play an important role in the pathogenesis of AD.

**Conclusion:**

The study aimed to provide a systematic understanding of the potential relationships among these genes and it is hoped that it could aid in AD biomarker discovery.

## Introduction

Aging causes an increasing susceptibility to cognitive performances due to a developing neurodegeneration leading to neurologic disorders, such as dementia. More than 20 million people worldwide suffer from dementia, and this number is expected to exceed 80 million by 2040 because of the rapid increase in the numbers of the elderly 1. Alzheimer’s Disease (AD) is an irreversible progressive neurodegenerative disease affecting the central nervous system. Despite the increasing rate of AD incidence, no therapeutic strategy has been developed yet 2. Pathophysiologically, AD-related brain severe shrinkage caused neural and synaptic degenerations 3. The mentioned degenerative events can be detected in post mortem examination of patients suffering from severe memory loss 4,5. It is thought that the loss of memory is because of aggregating beta amyloid (Aβ) and Neurofibrillary Tangles (NFTs) of hyper-phosphorylated tau protein 1,6. Additionally, inflammation characterized by activated microglia 7 and oxidative stress, which result from an imbalance of Reactive Oxygen Species (ROS) and antioxidants 8,9 were shown to be associated with AD. Epigenetic changes happening in pre-frontal region by aging were shown to be related with AD functioning at cognitive level 1.

Rewiring of the biological networks to detect co-regulated and co-expressed units will help to facilitate looking into network’s components and depicting the relationships between interconnected genes. Gene co-expression networks enable us to highlight molecular mechanisms underlying diseases 10 and are considered as one way to investigate the etiology of AD efficiently. A large number of co-expression network methods have been proposed in the literature 11,12. Differential Co-expression Analysis (DCEA) offers a powerful approach for exploring phenotypic changes 13. Not only is AD etiology incompletely understood but also differences at transcriptome level and the genes potentially related to each distinct regions of brain are not recognized causing AD to be remained somewhat unclear. In the present study, a high-throughput genomic screening approach was applied using DCGL software and comparative microarray analyses. It was hypothesized that the distinct transcriptional changes in different regions of brain lead to AD-associated brain damages. Therefore, the transcriptional profiles from the gray matter of frontal and temporal cortices were compared with hippocampi derived postmortem brains to dissect AD pathogenesis in these areas. The rationale behind the used network approach is to prioritize AD-causative genes that are apart from differential alterations in their expression and are differentially regulated by Transcription Factors (TFs) between contrasting samples. For this, Differential Regulation Analysis (DRA) has been conducted on three separated regions of AD brains as contrasting samples.

## Materials and Methods

### Data acquisition and pre-processing

The *CEL* *files* for GSE36980 series were downloaded from the GEO (http://www.ncbi.nlm.nih.gov/geo/) database and normalized with RMA method by using the Linear Models for Microarray Data (limma) R package. The main reason for selecting and exploiting this dataset is that GSE36980 series cover interspecies transcriptome analysis of various regions in gray matter in postmortem brains suiting the goal of dissecting pathological alterations in AD in several brain areas. Moreover, a number of researches have previously used these series and therefore would be able to compare the findings. After removing ambiguous probes, the extracted probe IDs were transformed into gene symbols. This data consists of a total of 79 samples (Table 1) based on the platform of GPL6244 and correspond to the frontal and temporal cortices and hippocampus.

**Table 1.** Sample characteristics

**Biological samples**	**Control**	**AD patients**
Temporal cortex	19	10
Frontal cortex	18	15
Hippocampus	10	7

### Network construction

The DCGL R package was used to conduct DCEA 13,14. This software firstly calculates Differential Co-expression profile (DCp) and Differential Co-expression enrichment (DCe) to extract significant co-expression changes between a pair of genes in control and treatment samples. Next, Differentially Co-expressed Genes (DCGs) and Differentially Co-expressed Links (DCLs) were summarized from DCp and DCe values.

Next, DCGs and DCLs were extracted from DCp and DCe values previously calculated by DCp and DCe functions. DCp filters co-expression values of a pair of genes were assessed in control and treatment conditions. *X* and *Y* were defined as a subset of the gene pairs, where *n* is co-expression neighbors for a gene;

*X =* (x_i_1, x_i_2, …, x_i_n)
*Y =* (y_i_1, y_i_2, …, y_i_n)

The DC of a given gene is calculated with the following equation:

$${\mathrm{DC}}_{n}(i)=\sqrt{\frac{{\left(x_{i1}-y_{i1}\right)}^{2}+{\left(x_{i2}-y_{i2}\right)}^{2}+\ldots +{\left(x_{in}-y_{in}\right)}^{2}}{n}}$$

If the resulting DCGs and DCLs coincide with a TF, they will be referred to as a DRG and DRL, respectively. The DRGs and DRLs were scrutinized by DR-sort function in Differential Regulation Analysis (DRA) module. In fact, DRA module identifies potential TF as upstream regulators of DCGs and DCLs 13. Finally, for illustrating the interactions between DRGs and their regulators, a network of DRGs and coincided TFs obtained by DRA was built for each of the datasets. By utilizing the Network Analyzer 15 nodes were set within networks with higher connections to darker color and bigger size.

### Gene ontology, pathway analysis and visualization

**Figure 1.**
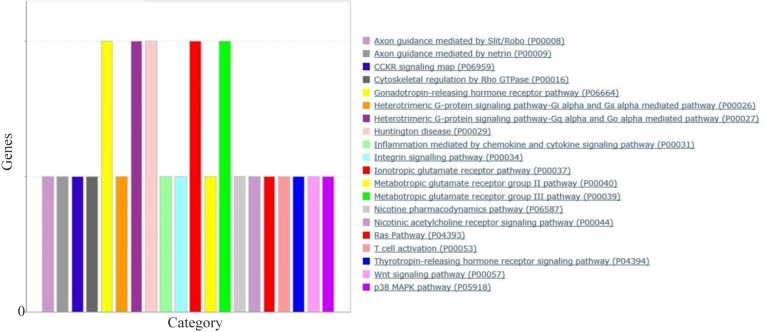
Bar chart of pathways potentially DRGs extracted from tempral cortex, frontal cortex and hippocampus expression data. PANTHER server with default parameters for pathway analysis was used for pathway analysis. The length of each bar showes how many genes have been assigned to a given pathway.

To find the significantly over-represented biological GO terms and functions of gene products within a co-expression network of DRGs and DRLs, functional classification was performed using BINGO Cytoscape plugin 16 running hypergeometric test and Benjamini & Hochberg FDR correction at significant level 0.01. Finally, the clusters were visualized by Enrichment map Cytoscape plugin with Jaccard’s coefficient 0.001. DRGs were further functionally classified by PANTHER database (http://pantherdb.org/) to underlying pathways (Figure 1).

## Results

### Co-expression analysis

**Figure 2.**
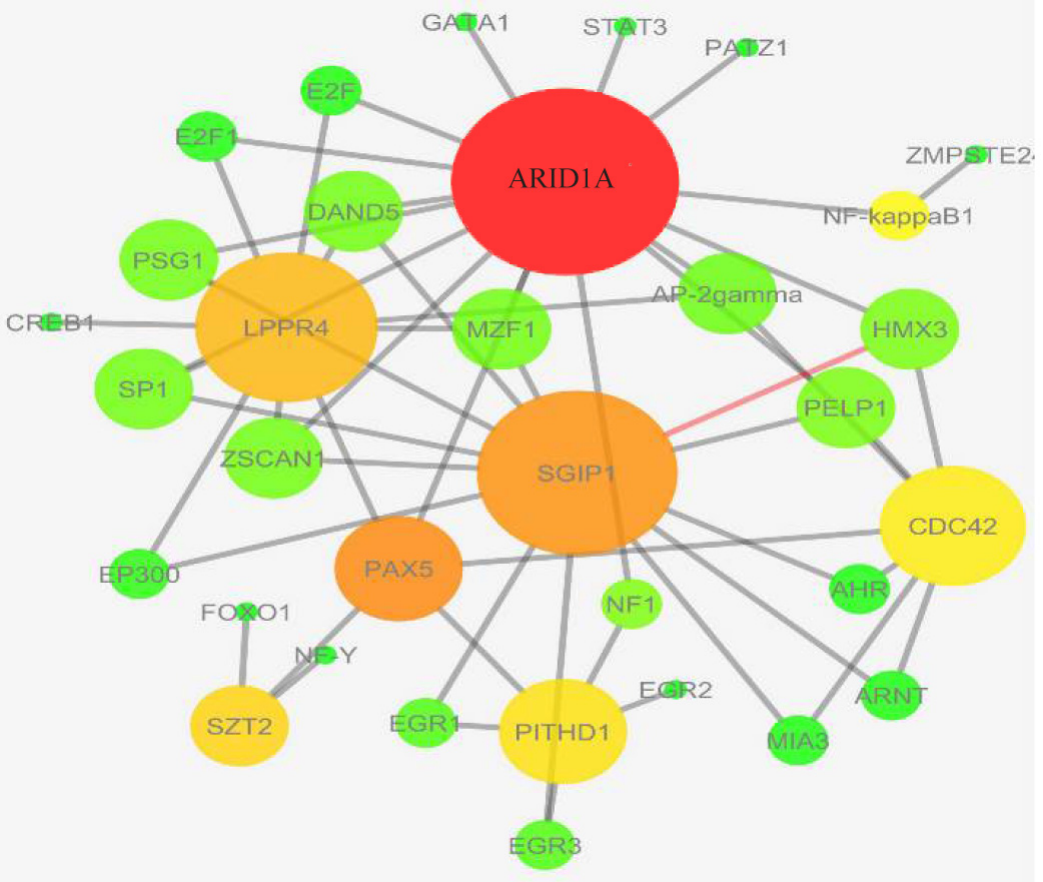
Differential co-expressed network of DRGs and DRLs captured by TED and TDD results in temporal cortex datasets. The bigger and darker nodes show the nodes with higher connectivity within the network.

**Figure 3.**
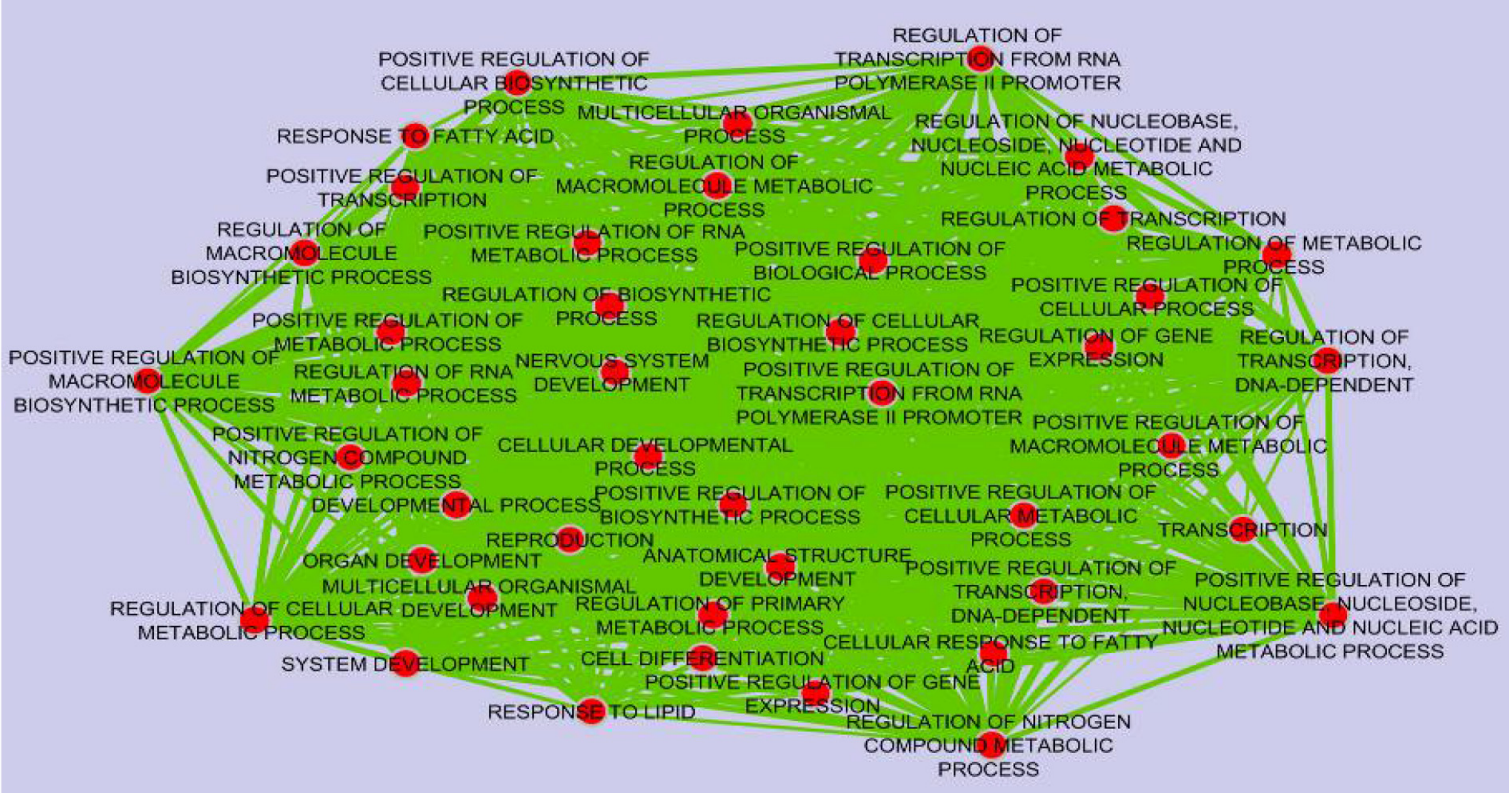
Functional classification of biological process in which Differential Regulated Genes (DRGs) were supposed to be involved. The GO terms were considered significant based on hypergeometric test with Benjamini & Hochberg FDR correction and significance level 0.01 by BINGO app. The results were illustrated using the Enrichment map Cytoscape 3.4.0 plugin. Ticker lines and bigger circles show more genes with higher significance level belonging to a given term.

The expression values of GSE36980 datasets were analyzed by utilizing DCGL v2.0 R package with default parameters. A total of 17088 unique genes were subjected to expression based filter and variance based filter, two functions embedded in DCGL to filter out genes that expressed extreme invariability across control and AD samples yielding 8544 and 2918 genes, respectively (Supplementary file 1). Afterward, using 2918 unique genes, co-expression analysis was performed on temporal cortex, frontal cortex, and hippocampus datasets separately. Expression based filter removes genes whose mean expression between experiments is lower than the median of this value for all genes and variance based filter removes genes that are not significantly variable than the median gene 13. In order to prioritize seed genes which are potentially related to AD pathogenesis, common and significant DRGs were selected using Targets’ Enrichment Density (TED) analysis and Targets’ DCL Density (TDD) analysis. TED and TDD identify differential co-expression genes and link in a particular TF’s targets, respectively 13. To this end, targets of significant TFs were extracted from 19,9950 TF-to-target interaction pairs as a library in DCGL v2.0 software 13. These pairs were further filtered out based on DRLs. In sum, 7, 19 and 13 genes were identified in temporal cortex, frontal cortex and hippocampus, respectively (Table 2). Significant TFs derived by TED and TDD analysis were used to infer co-expression network of DRGs in each dataset independently (Figure 2, Supplementary figures). DRGs were classified in terms of response to lipid, response to fatty acid, regulation of transcription from RNA polymerase II promoters and regulation of nitrogen compound metabolic process (Figure 3). Moreover, in pathway analysis, signaling pathways such as glutamate and G-protein signaling pathways were noteworthy (Figure 1).

**Table 2.** List of differential regulated genes (DRGs) and corresponding p-value<0.05 of differential co-expression enrichment (DCe) in temporal cortex, frontal cortex and hippocampus datasets

	**Gene name**	**DCe p-value**	**Description**
**Temporal cortex**	*ARID1A*	0.00069	*ARID1A* was among down-regulated genes in AD model mice (17)
	*Cdc42*	0.01224	*Cdc42* activity was increased in hippocampus neurons treated with fibrillary β-amyloid (18)
	*LPPR4*	0.01395	LPPR4 was up-regulated in incipient AD patients (19)
	*PITHD1*	0.01863	//
	*SGIP1*	0.02064	//
	*SZT2*	0.01936	//
	*ZMPSTE24*	0.01936	//

**Frontal cortex**	*CHD5*	5.19E-14	The depletion of *CHD5* was shown to be linked with AD associated gene sets (20)
	*EFHD2*	3.00E-07	*EFhd2* has been found to be associated with aggregated tau in the brain in AD and in a mouse model of frontotemporal dementia (21,22)
	*Prxs*	7.82E-07	Peroxiredoxins (Prxs) may be associated with AD by reducing ROS elicited by amyloid β (Aβ) accumulation that could be a causative factor in the pathogenesis of AD (23)
	*MAGIE3*	1.68E-06	//
	*EXTL1*	4.14E-06	//
	*HPCAL4*	2.09E-05	*HPCAL4* could be used as a prognostic marker for cognitive decline in AD (24)
	*LPHN2*	2.59E-05	*LPHN2* is likely to be participated in AD as an altered protein in Lipid Raft (25)
	*NIPAL3*	8.35E-05	*NIPAL3* was shown as a biomarker in Late-Onset Major Depressive Disorder (26)
	*CACNA1E*	0.00017	*CACNA1E* was down-regulated in cerebral Cockayne syndrome (27)
	*IFI16*	0.00033	IFI16 was participated in delaying onset of AD (28)
	*HHLA3*	0.00122	//
	*KCNK1*	0.00202	*KCNK1* exhibited alternative splicing in patients with mesial temporal lobe epilepsy (29)
	*rnpc3*	0.00384	//
	*DCAF6*	0.00542	//
	*IPO13*	0.00581	*IPO13* mutants involved in chronic inflammatory diseases (30)
	*RPL11*	0.00585	*RPL11* revealed significant altered expression profiles in the neuron model of AD treated with rhTFAM (31)
	*S100A1*	0.00716	*S100A1* modulates inflammation in AD (32)
	*CNTN2*	0.02606	*CNTN2* associated with AD via *BACE1* activity (33)
	*GRIK3*	0.03774	*GRIK3* was highly expressed in major depression (34)

**Hippocampus**	*KCNK1*	1.55E-09	//
	*CHRNB2*	2.37E-09	*CHRNB2* was found to interfere with the immune system in neurologic disorders (35)
	*HAPLN2*	3.43E-05	*Hapln2* has been recently shown to be accumulated in the neurofibrillary tangle of Alzheimer’s brain (36)
	**Slc2a1**	0.00207	**Slc2a1* down-regulation exacerbated AD (37)*
	*FABP3*	0.00298	serum levels of brain-type *FABP* are elevated in a significant proportion of patients with various neurodegenerative diseases including AD (38)
	*DEGS1*	0.00327	*DEGS1* is likely to be involved in AD as an altered protein in Lipid Raft (25)
	*NKAIN1*	0.00435	//
	*S100A1*	0.00434	*S100A1* modulates inflammation in AD (32)
	*CNTN2*	0.00511	*CNTN2* associated with AD via b-Secretase (*BACE1*) activity (33)
	*SFPQ*	0.00816	*SFPQ* was shown as a transcription factor with an altered nucleo-cytoplasmic distribution under neurodegenerative conditions (39)
	*GPSM2*	0.01073	//
	*GSTM1*	0.01591	*GSTM1* null genotype was found as risk factor for late-onset Alzheimer’s disease in Italian patients (40)
	*CACHD1*	0.02830	*CACHD1* is a substrate of *BACE1* responsible for generating the amyloid-b protein (41)

// showing DRGs with ambiguous role in neurologic disorders.

### Temporal cortex

460 DCGs and 33656 DCLs were summarized using DC sum function to a final set of DCGs and DCLs (Supplementary file 2). There were 199 significant TFs in the results of TED analysis and 35 significant TFs in TDD analysis. 35 TFs that were significant in both of these two analysis results were chosen (Supplementary file 2). DRA analysis yielded 7 DRGs and 33 DRLs. DRGs were not only differentially co-expressed but also differentially co-regulated with 35 mentioned TFs. Then, a network of DRGs and DRLs was visualized using Cytoscape 3.4.0. Based on figure 2, *PAX5* transcription factor and genes including *ARID1A*, *CDC42* and LPPR4 were highlighted as the most important units within the genes network with more interconnected links (Figure 2).

### Frontal cortex

In frontal cortex datasets, 628 DCGs and 166256 DCLs were summarized to 20 DRGs and 164 DRLs (Supplementary file 3). There were 199 significant TFs in TED analysis result and 135 significant TFs in TDD analysis result from which 135 TFs were chosen that were significant in both TED and TDD results (Supplementary file 3). In the inferred network, *PAX5* and *IKZF1* as TFs and genes including *GRIK3*, *MAGI3*, *PRRX1* and *DCAF6* were found as highlighted nodes with more connectivity.

### Hippocampus

According to hippocampus datasets, 670 DCGs and 56264 DCLs were summarized to 16 DRGs and 43 DRLs (Supplementary file 4). There were 199 common and significant TFs in TED and TDD analysis which were used for inferring differential co-expression network with DRGs. There was more connectivity in hippocampus network than the other two networks. *PAX5*, *ARNT*, *GATA1*, *EGR3* and *IKZF1* TFs and genes including *KCNK1*, *CACHD1*, *FABP3* and *CHRNB2* showed highlighted roles as network nodes.

## Discussion

Aging is believed to be one of the most important non-modifiable risk factors of cognitive diseases that lead unequivocally to a number of detrimental changes in the neural system, increasing neuromorbidity and mortality. AD, as a progressive neurodegenerative disorder with no effective treatment options, is typically characterized by the presence of amyloid-beta plaques and hyper phosphorylated paired helical filament tau protein-rich neurofibrillary tangles 1. The identification of co-expressed genes related to AD presumably provides insights into the underlying mechanisms; in other words, a combination of gene effects likely holds promise as a more effective approach for detecting disease associated genes 42. In fact, examining co-expressed genes in spite of the individual genes could be more informative to explore genes that cause mental health disorders, such as AD 43,44. In this case, the correlation between two genes varies in distinct samples and thereby they are referred to as being differentially co-expressed. This correlation may change independently from the expression levels of two genes, indicating that transcriptome analysis merely based on differential expression analysis could miss important clues of regulatory patterns 45. Co-expression analysis has been performed for deciphering molecular mechanisms underlying mental health disorders 46–49. In the context of a well-established network analysis approach and given the most variable transcripts between control and AD brain samples, attempts were made by DCGL framework to explore putative pivotal genes that may be associated with AD. This work attempted to identify DRGs and links DRLs in AD by comparing expression datasets of temporal and frontal cortices and hippocampi. A comprehensive search in the literature showed that the obtained DRGs of AD brains mostly have direct or in-direct links with AD or another neurologic disorder (Table 2). They are implicated in the gene ontology terms and shared biological pathways like response to lipid, fatty acid, nitrogen compound metabolic process and glutamate signaling pathways (Figures 1 and 2, supplementary file 5). Reportedly, considering GO terms as the response to lipid and fatty acid, brain lipid homeostasis plays an important role in AD 50. In this regard, differential regulation of delta 4-desaturase, sphingolipid 1 (*DEGS1*) and fatty acid binding protein 3 (*FABP3*) in hippocampus and lipid phosphate phosphatase-related protein type 4 (*LPPR4*) in temporal cortex datasets may fairly explain the relationship between brain damages happening in these regions and lipid metabolism. *DEGS1* encodes a member of the membrane fatty acid desaturase family which is shown to interfere in AD via lipid rafts 25. FABP proteins are thought to participate in the uptake, intracellular metabolism and/or transport of long-chain fatty acids. Concordantly, serum levels of brain-type *FABP* are elevated in a significant proportion of patients with various neurodegenerative diseases including AD 38. *LPPR4* acts as phospholipid dephosphorylate involving axonogenesis. The control of ion flow across the lipid membrane is essential for many cellular functions, including neuronal excitability and dysfunction of conveying ions through lipid bilayers involved in multiple neurologic diseases 51. As illustrated in figure 1, the DRGs are more implicated in signaling pathways; but the DRGs from frontal cortex were more enriched in ionotropic glutamate receptor pathway and metabo-tropic glutamate receptor group II and III pathways. The dysregulation of glutamatergic signaling has been shown to be associated with AD. Glutamate acts *via* ionotropic glutamate receptors (*iGluR*) and metabo-tropic glutamate receptors (*mGluR*), both of which have been implicated in AD 52. Differential regulation of glutamate receptor ionotropic, kainate 3 (*GRIK3*) and voltage-dependent R-type calcium channel subunit alpha-1E (*CACNA1E*) in frontal cortex datasets may be biologically relevant with the mentioned pathways in AD brain areas. Concordantly, a significant change in the expression of the *GRIK3* gene was detected in a patient diagnosed with severe developmental delay 53. Many different kinds of signaling pathways are changed in AD, indeed the relevance of the biological pathways shown in figure 1 such as cytoskeletal regulation by Rho GTPase suggests mediating of these signaling pathways in the different lobs of brain, in this case in temporal cortex with differential regulation of *CDC42*. *CDC42* has been linked to neuronal diseases like Alzheimer and Parkinson’s disease through its role in cytoskeletal organization 54. Among the DGRs, *CNTN2*, *KCNK1* and *S100A1* were found common in frontal cortex and hippocampus datasets. S100A1 encodes for calmodulin signaling molecules. Increased levels of calmodulin have been reported in the hippocampus of AD model mice 55. These changes seemingly show an aberrant involvement of calmodulin in the impairment of cell cycle control in AD. As for the potassium channel subfamily K member 1- *KCNK1*, recent genetic studies suggest a central role for neuroinflammation. *KCNK1* is a voltage-gated potassium channel upregulated by activated microglia and a mediator in amyloid-mediated microglial priming, additionally reactive oxygen species production that was shown to be related with autoimmunity 56. CNTN2 has been shown to undergo nuclear translocation and altered transcription 33.

These findings probably show that hippocampus and frontal cortices might deeply play a role in AD by mediating with conveying ions. Their obtained DRGs participated in vital processes like signaling, ion transportation and homeostasis. However, these processes mostly signal pathways somehow shared with temporal cortex implying the role of signal molecules within and between brain areas in neurologic dysfunctions. Concordantly, a comprehensive study has been already carried out on GSE36980 series to examine the alteration in the expression of diabetes-related genes in AD brains where they illustrated that hippocampi of AD brains have the most significant alteration in gene expression profile 57.

With a glance at table 2 and the terms including amyloids, inflammation, ROS and immune system, one could infer a cascade of events in which the DRGs interfere. Beta-amyloid deposition following the activation of microglia will initiate an inflammatory response leading to the release of potentially neurotoxic substances and ROS that targets neural damage 58. Afterward, along with immune response, nitrogen compounds will mediate to reverse the consequences of oxidative stress in damaged regions 8,9. In sum, it was shown that DRGs covered a wide range of known functions and processes implicated in main AD signaling pathways. In a study by Satoh *et al* 59, GSE36980 series used in the present study were utilized to identify biomarker genes relevant to the molecular pathogenesis of AD. They analyzed a RNA-Seq dataset composed of the transcriptome of postmortem AD brains derived from two independent cohorts and they identified the core set of 522 genes deregulated in AD brains shared between both, compared with normal control subjects. Notably, in agreement with our study, LPPR4 was bolded in AD brains in both microarray and RNA-seq datasets. By consistent downregulation of NeuroD6 in AD brains, the results indicated that downregulation of NeuroD6 serves as a possible biomarker for AD brains. Previous studies identified LPPR4 as direct target genes for NeuroD6 by binding assay to E-boxes located in target gene promoters 60. GSE36980 series were also employed by Fowler *et al* 61 used to investigate potential underlying biology in AD and in concordance with the results of the present study, they noticed the overrepresentation of glutamate in their data mining. They first identified genes consistently associated with AD in each of the four separate expression studies, and confirmed the result using a fifth study. They next developed algorithms to search hundreds of thousands of GEO data sets, identifying a link between an AD-associated gene (NEUROD6) and gender. Additionally, they identified several genes related to glutamate (including CACNG3, a regulator of AMPA-sensitive glutamate receptors; SLC17A7, a mitochondrial oxoglutarate carrier; and GOT2, mitochondrial glutamic-oxaloacetic transaminase. In our study, differential regulation of glutamate receptor ionotropic, kainate 3 (GRIK3) and voltage-dependent R-type calcium channel subunit alpha-1E (CACNA1E) in frontal cortex datasets could be therefore biologically relevant with the mentioned pathways in AD brain areas. Moreover, in our study, differential regulation of Slc2a1 in hippocampus data seemingly implies the role of impairments in glutamatergic transmission mostly in hippocampus of AD brains. The role of glutamate transporters such as SLC1A6 was also highlighted in a study by Satoh *et al* 59.

## Conclusion

The purpose of the study was to explore the molecular mechanism in the development of AD, and a comparison of AD in three regions of the brain was done. Therefore, in the frame of network reconstruction and data mining approaches, a small number of possible genes and TFs were identified that their interplay could lead to neural dysfunctions toward AD. However, one should be cautious regarding small sample size while by utilizing more adequate samples, the results would be more reliable evidences.

An expected outcome of such a work would possibly shed light on the bridges between AD-associated brain damage in transcriptome level and presenting crucial evidence in clinical diagnosis and treatment.

## References

[1] Ferri CP Prince M Brayne C Brodaty H Fratiglioni L Ganguli M Global prevalence of dementia: a Delphi consensus study. Lancet 2005;366(9503):2112–2117. 16360788 10.1016/S0140-6736(05)67889-0 PMC2850264

[2] Alzheimer's Association 2013 Alzheimer's disease facts and figures. Alzheimer's Dement 2013;9(2):208–245. 23507120 10.1016/j.jalz.2013.02.003

[3] Dubois B Feldman HH Jacova C Hampel H Molinuevo JL Blennow K Advancing research diagnostic criteria for Alzheimer's disease: the IWG-2 criteria. Lancet Neurol 2014;13(6):614–629. 24849862 10.1016/S1474-4422(14)70090-0

[4] Alzheimer A. Ueber eine eigenartige erkrankung der hirnrinde. Z Psychiatr 1907;64:146–148.

[5] Alzheimer A Stelzmann RA Schnitzlen HN Murthagh FR. An english translation of alzheimer's 1907 paper, “Uber eine eigenartige erkankung der hirnrinde”. Clin Anat 1995;8(6):429–431. 8713166 10.1002/ca.980080612

[6] Braak E Braak H. Alzheimer's disease: transiently developing dendritic changes in pyramidal cells of sector CA1 of the Ammon's horn. Acta Neuropathol 1997;93(4):323–325. 9113196 10.1007/s004010050622

[7] Glass CK Saijo K Winner B Marchetto MC Gage FH. Mechanisms underlying inflammation in neurodegeneration. Cell 2010;140(6):918–934. 20303880 10.1016/j.cell.2010.02.016 PMC2873093

[8] Varadarajan S Yatin S Aksenova M Butterfield DA. Review: Alzheimer's amyloid beta-peptide-associated free radical oxidative stress and neurotoxicity. J Struct Biol 2000;130(2–3):184–208. 10940225 10.1006/jsbi.2000.4274

[9] Guglielmotto M Giliberto L Tamagno E Tabaton M. Oxidative stress mediates the pathogenic effect of different Alzheimer's disease risk factors. Front Aging Neurosci 2010;2:3. 20552043 10.3389/neuro.24.003.2010 PMC2874401

[10] Gaiteri C Ding Y French B Tseng GC Sibille E. Beyond modules and hubs: the potential of gene co-expression networks for investigating molecular mechanisms of complex brain disorders. Genes Brain Behav 2014;13(1):13–24. 24320616 10.1111/gbb.12106 PMC3896950

[11] Stoner R Chow ML Boyle MP Sunkin SM Mouton PR Roy S Patches of disorganization in the neocortex of children with autism N Engl J Med 2003;370(13):1209–1219. 10.1056/NEJMoa1307491 PMC4499461 24670167

[12] Jinawath N Bunbanjerdsuk S Chayanupatkul M Ngamphaiboon N Asavapanumas N Svasti J Bridging the gap between clinicians and systems biologists: from network biology to translational biomedical research. J Transl Med 2016;14(1):324. 27876057 10.1186/s12967-016-1078-3 PMC5120462

[13] Yang J Yu H Liu BH Zhao Z Liu L Ma LX DCGL v2.0: an R package for unveiling differential regulation from differential co-expression. PLoS One 2013;8(11):e79729. 10.1371/journal.pone.0079729 PMC3835854 24278165

[14] Liu BH Yu H Tu K Li C Li YX Li YY. DCGL: an R package for identifying differentially co-expressed genes and links from gene expression microarray data. Bioinformatics 2010;26(20):2637–2638. 20801914 10.1093/bioinformatics/btq471 PMC2951087

[15] Assenov Y Ramirez F Schelhorn SE Lengauer T Albrecht M. Computing topological parameters of biological networks. Bioinformatics 2008;24(2):282–284. 18006545 10.1093/bioinformatics/btm554

[16] Maere S Heymans K Kuiper M. BiNGO: a Cytoscape plugin to assess overrepresentation of gene ontology categories in biological networks. Bioinformatics 2005;21(16):3448–3449. 15972284 10.1093/bioinformatics/bti551

[17] Gatta V D'Aurora M Granzotto A Stuppia L Sensi SL. Early and sustained altered expression of aging-related genes in young 3xTg-AD mice. Cell Death Dis 2014; 5:e1054. 10.1038/cddis.2014.11 PMC3944230 24525730

[18] Mendoza-Naranjo A Gonzalez-Billault C Maccioni RB. Abeta1-42 stimulates actin polymerization in hippocampal neurons through Rac1 and Cdc42 Rho GTPases. J Cell Sci 2007;120(Pt 2):279–288. 17200137 10.1242/jcs.03323

[19] Williams C Mehrian Shai R Wu Y Hsu YH Sitzer T Spann B Transcriptome analysis of synaptoneurosomes identifies neuroplasticity genes overexpressed in incipient Alzheimer's disease. PLoS One 2009;4(3):e4936. 10.1371/journal.pone.0004936 PMC2654156 19295912

[20] Potts RC Zhang P Wurster AL Precht P Mughal MR Wood WH 3rd CHD5, a brain-specific paralog of Mi2 chromatin remodeling enzymes, regulates expression of neuronal genes. PLoS One 2011;6(9):e24515. 10.1371/journal.pone.0024515 PMC3172237 21931736

[21] Borger E Herrmann A Mann DA Spires-Jones T Gunn-Moore F. The calcium-binding protein EFhd2 modulates synapse formation in vitro and is linked to human dementia. J Neuropathol Exp Neurol 2014;73(12):1166–1182. 25383639 10.1097/NEN.0000000000000138 PMC4238966

[22] Vega IE. EFhd2, a protein linked to Alzheimer's disease and other neurological disorders. Front Neurosci 2016; 10:150. 27064956 10.3389/fnins.2016.00150 PMC4814571

[23] Cumming RC Dargusch R Fischer WH Schubert D. Increase in expression levels and resistance to sulfhydryl oxidation of peroxiredoxin isoforms in amyloid beta-resistant nerve cells. J Biol Chem 2007;282(42):30523–30534. 17761673 10.1074/jbc.M700869200

[24] Braunewell KH. The visinin-like proteins VILIP-1 and VILIP-3 in Alzheimer's disease-old wine in new bottles. Front Mol Neurosci 2012;5:20. 22375104 10.3389/fnmol.2012.00020 PMC3284765

[25] Chadwick W Brenneman R Martin B Maudsley S. Complex and multidimensional lipid raft alterations in a murine model of Alzheimer's disease. Int J Alzheimers Dis 2010;2010:604792. 10.4061/2010/604792 PMC2997345 21151659

[26] Miyata S Kurachi M Okano Y Sakurai N Kobayashi A Harada K Blood transcriptomic markers in patients with late-onset major depressive disorder. PLoS One 2016;11(2):e0150262. 10.1371/journal.pone.0150262 PMC4771207 26926397

[27] Wang Y Cella M Mallinson K Ulrich JD Young KL Robinette ML TREM2 lipid sensing sustains microglia response in an Alzheimer's disease model. Cell 2015;160(6):1061–1071. 25728668 10.1016/j.cell.2015.01.049 PMC4477963

[28] Vélez JI Lopera F Sepulveda-Falla D Patel HR Johar AS Chuah A. APOE*E2 allele delays age of onset in PSEN1 E280A Alzheimer's disease. Mol Psychiatry 2016;21(7):916–924. 26619808 10.1038/mp.2015.177 PMC5414071

[29] Heinzen EL Yoon W Weale ME Sen A Wood NW Burke JR Alternative ion channel splicing in mesial temporal lobe epilepsy and Alzheimer's disease. Genome Biol 2007;8(3):R32. 17343748 10.1186/gb-2007-8-3-r32 PMC1868939

[30] De Iudicibus S Franca R Martelossi S Ventura A Decorti G. Molecular mechanism of glucocorticoid resistance in inflammatory bowel disease. World J Gastroenterol 2011;17(9):1095–1108. 21448414 10.3748/wjg.v17.i9.1095 PMC3063901

[31] Oka S Leon J Sakumi K Ide T Kang D LaFerla FM Human mitochondrial transcriptional factor a breaks the mitochondria-mediated vicious cycle in Alzheimer's disease. Sci Rep 2016;6:37889. 10.1038/srep37889 PMC5126576 27897204

[32] Zimmer DB Keeling D Campbell K Campbell K Afanador L. S100A1 modulates inflammation and PI3/Akt signaling. FASEB J 2013;27(1Suppl):lb516.

[33] Gautam V D'Avanzo C Hebisch M Kovacs DM Kim DY. BACE1 activity regulates cell surface contactin-2 levels. Mol Neurodegener 2014;9:4. 24405708 10.1186/1750-1326-9-4 PMC3899608

[34] Gray AL Hyde TM Deep-Soboslay A Kleinman JE Sodhi MS. Sex differences in glutamate receptor gene expression in major depression and suicide. Mol Psychiatry 2015;20(9):1139. 26216299 10.1038/mp.2015.114

[35] Olgiati P Politis AM Papadimitriou GN Ronchi DD Serretti A. Genetics of late-onset Alzheimer's disease: update from the alzgene database and analysis of shared pathways. Int J Alzheimers Dis 2011;2011:832379. 10.4061/2011/832379 PMC3235576 22191060

[36] Bandopadhyay R. Sequential extraction of soluble and insoluble alpha-synuclein from parkinsonian brains. J Vis Exp 2016;(107). 10.3791/53415 PMC4781043 26780369

[37] Winkler EA Nishida Y Sagare AP Rege SV Bell RD Perlmutter D GLUT1 reductions exacerbate Alzheimer's disease vasculoneuronal dysfunction and degeneration. Nat Neurosci 2015;18(4):521–530. 25730668 10.1038/nn.3966 PMC4734893

[38] Teunissen CE Veerhuis R De Vente J Verhey FR Vreeling F van Boxtel MP Brain-specific fatty acid-binding protein is elevated in serum of patients with dementia-related diseases. Eur J Neurol 2011;18(6):865–871. 21143341 10.1111/j.1468-1331.2010.03273.x

[39] Ke Y Dramiga J Schütz U Kril JJ Ittner LM Schröder H Tau-mediated nuclear depletion and cytoplasmic accumulation of SFPQ in Alzheimer's and Pick's disease. PLoS One 2012;7(4):e35678. 10.1371/journal.pone.0035678 PMC3338448 22558197

[40] Piacentini S Polimanti R Squitti R Ventriglia M Cassetta E Vernieri F GSTM1 null genotype as risk factor for late-onset Alzheimer's disease in Italian patients. J Neurol Sci 2012;317(1–2):137–140. 22381228 10.1016/j.jns.2012.01.026

[41] Hemming ML Elias JE Gygi SP Selkoe DJ. Identification of beta-secretase (BACE1) substrates using quantitative proteomics. PLoS One 2009;4(12):e8477. 10.1371/journal.pone.0008477 PMC2793532 20041192

[42] Xu Y Yue W Shugart YY Li S Cai L Li Q Exploring transcription factors-microRNAs Co-regulation networks in Schizophrenia. Schizophr Bull 2016;42(4):1037–1045. 26609121 10.1093/schbul/sbv170 PMC4903044

[43] Oldham MC Horvath S Geschwind DH. Conservation and evolution of gene co-expression networks in human and chimpanzee brains. Proc Natl Acad Sci USA 2006; 103(47):17973–17978. 17101986 10.1073/pnas.0605938103 PMC1693857

[44] Okamura Y Aoki Y Obayashi T Tadaka S Ito S Narise T COXPRESdb in 2015: coexpression database for animal species by DNA- microarray and RNAseq-based expression data with multiple quality assessment systems. Nucleic Acids Res 2015;43(Database issue):D82–86. 25392420 10.1093/nar/gku1163 PMC4383961

[45] de la Fuente A. From ‘differential expression’ to ‘differential networking’ - identification of dysfunctional regulatory networks in diseases. Trends Genet 2010;26(7):326–333. 20570387 10.1016/j.tig.2010.05.001

[46] Torkamani A Dean B Schork NJ Thomas EA. Coexpression network analysis of neural tissue reveals perturbations in developmental processes in schizophrenia. Genome Res 2010;20:403–412. 20197298 10.1101/gr.101956.109 PMC2847743

[47] de Jong S Boks MP Fuller TF Strengman E Janson E de Kovel CG A gene co-expression network in whole blood of schizophrenia patients is independent of antipsychotic-use and enriched for brain-expressed genes. PLoS One 2012;7(6):e39498. 10.1371/journal.pone.0039498 PMC3384650 22761806

[48] Ponomarev I Wang S Zhang L Harris RA Mayfield RD. Gene co-expression networks in human brain identify epigenetic modifications in alcohol dependence. J Neurosci 2012;32(5):1884–1897. 22302827 10.1523/JNEUROSCI.3136-11.2012 PMC3564514

[49] Chen C Cheng L Grennan K Pibiri F Zhang C Badner JA Two gene co-expression modules differentiate psychotics and controls. Mol Psychiatry 2013;18(12):1308–1314. 23147385 10.1038/mp.2012.146 PMC4018461

[50] El Gaamouch F Jing P Xia J Cai D. Alzheimer's disease risk genes and lipid regulators. J Alzheimers Dis 2016;23;53(1):15–29. 27128373 10.3233/JAD-160169

[51] Yuan H Low CM Moody OA Jenkins A Traynelis SF. Ionotropic GABA and Glutamate receptor mutations and human neurologic diseases. Mol Pharmacol 2015;88(1):203–217. 25904555 10.1124/mol.115.097998 PMC4468639

[52] Hamilton A Zamponi GW Ferguson SS. Glutamate receptors function as scaffolds for the regulation of β-amyloid and cellular prion protein signaling complexes. Mol Brain 2015;8:18. 25888324 10.1186/s13041-015-0107-0 PMC4395978

[53] Takenouchi T Hashida N Torii C Kosaki R Takahashi T Kosaki K. 1p34.3 deletion involving GRIK3: further clinical implication of GRIK family glutamate receptors in the pathogenesis of developmental delay. Am J Med Genet A 2014;164A(2):456–460. 24449200 10.1002/ajmg.a.36240

[54] Schnack C Danzer KM Hengerer B Gillardon F. Protein array analysis of oligomerization-induced changes in alpha-synuclein protein-protein interactions points to an interference with Cdc42 effector proteins. Neuroscience 2008;154(4):1450–1457. 18541383 10.1016/j.neuroscience.2008.02.049

[55] Min D Guo F Zhu S Xu X Mao X Cao Y The alterations of Ca2+/calmodulin/CaMKII/CaV1.2 signaling in experimental models of Alzheimer's disease and vascular dementia. Neurosci Lett 2013;538:60–65. 23403102 10.1016/j.neulet.2013.02.001

[56] Rangaraju S Gearing M Jin LW Levey A. Potassium channel Kv1.3 is highly expressed by microglia in human Alzheimer's disease. J Alzheimers Dis 2015;44(3):797–808. 25362031 10.3233/JAD-141704 PMC4402159

[57] Hokama M Oka S Leon J Honda H Sasaki K Nakabeppu Y Altered expression of diabetes-related genes in Alzheimer's disease brains: the Hisayama study. Cereb Cortex 2014;24(9):2476–2488. 23595620 10.1093/cercor/bht101 PMC4128707

[58] Rogers J. The inflammatory response in Alzheimer's disease. J Periodontol 2008;79(8 Suppl):1535–1543. 18673008 10.1902/jop.2008.080171

[59] Satoh J Yamamoto Y Asahina N Kitano S Kino Y. RNA-Seq data mining: downregulation of neuroD6 serves as a possible biomarker for Alzheimer's disease brains. Dis Markers 2014;2014:123165. 10.1155/2014/123165 PMC4274867 25548427

[60] Yamada M Shida Y Takahashi K Tanioka T Nakano Y Tobe T Prg1 is regulated by the basic helix-loop-helix transcription factor Math2. J Neurochem 2008;106(6):2375–2384. 18643870 10.1111/j.1471-4159.2008.05579.x

[61] Fowler KD Funt JM Artyomov MN Zeskind B Kolitz SE Towfic F. Leveraging existing data sets to generate new insights into Alzheimer's disease biology in specific patient subsets. Sci Rep 2014;5:14324. 10.1038/srep14324 PMC4585817 26395074

